# Construction of a miRNA Signature Using Support Vector Machine to Identify Microsatellite Instability Status and Prognosis in Gastric Cancer

**DOI:** 10.1155/2022/6586354

**Published:** 2022-04-15

**Authors:** Ya-nan Wang, Ya-ning Wei, Xiang-yu Fan, Fang Xu, Zheng-yang Dong, Shu-jie Cheng, Jin-ku Zhang

**Affiliations:** ^1^Department of Pathology, Affiliated Hospital of Hebei University, Baoding, China; ^2^Department of Oncology, Affiliated Hospital of Hebei University, Baoding, China; ^3^Department of Pathology, The Fourth Hospital of Hebei Medical University, Shijiazhuang, China; ^4^Department of Surgery, Affiliated Hospital of Hebei University, Baoding, China; ^5^Department of Pathology, No.1 Central Hospital of Baoding, Baoding, China

## Abstract

**Background:**

The specific role and prognostic value of DNA repair and replication-associated miRNAs in gastric cancer (GC) have not been clearly elucidated. Therefore, comprehensive analysis of miRNAs in GC is crucial for proposing therapeutic strategies and survival prediction.

**Methods:**

Firstly, clinical information and transcriptome data of TCGA-GC were downloaded from the database. In the entire cohort, we performed differential analysis in all miRNAs and support vector machine (SVM) was used to eliminate redundant miRNAs. Subsequently, we combined survival data and cox regression analysis to construct a miRNA signature in the training cohort. In addition, we used PCA, Kaplan-Meier, and ROC analysis to explore the prognosis value of risk score in the training and testing cohort. It is worth noting that multiple algorithms were used to evaluate difference of immune microenvironment (TME), microsatellite instability (MSI), tumor mutational burden (TMB), and immunotherapy in different risk groups. Finally, we investigated the potential mechanism about miRNA signature.

**Results:**

We constructed miRNA signature based on the following 4 miRNAs: hsa-miR-139-5p, hsa-miR-139-3p, hsa-miR-146b-5p, and hsa-miR-181a-3p. Univariate and multivariate Cox regression analyses suggested that risk score is a risk factor and an independent prognostic factor in GC patients. The AUC value of ROC analysis showed a robust prediction accuracy in each cohort. Moreover, significant differences in immune functions, immune cell content, immune checkpoint, MSI status, and TMB score were excavated in different groups distinguished by risk score. Finally, based on the above four miRNA target genes, we revealed that the signature was enriched in DNA repair and replication.

**Conclusion:**

We have developed a robust risk-formula based on 4 miRNAs that provides accurate risk stratification and prognostic prediction for GC patients. In addition, different risk subgroups may potentially guide the choice of targeted therapy.

## 1. Introduction

Gastric cancer (GC) is one of the most common causes of death across the world [1]. Its overall 5-year survival rate is less than 20%, and although considerable progress has been made in the treatment of GC, only slight improvements have been seen in the past 20 years [2]. Microsatellite instability (MSI) increases the rate of replication mistakes and hypermutation state, increasing the risk of oncogene or tumor suppressor gene alterations. Importantly, MSI status in patients with GC has been proved to be useful for treatment outcome prediction [3]. However, research combining several miRNAs to predict MSI status and prognosis is relatively uncommon.

Meanwhile, miRNAs are endogenous noncoding RNAs ranging in length from 17 to 25 nucleotides that influence gene expression posttranscriptionally [4]. Increasing data indicates that miRNA expression is varied in GC and is linked with survival prognosis [5]. Numerous research published in the last few years have identified miRNAs as possible diagnostic or prognostic indications for GC; however, the findings have been conflicting, although recent studies suggest that miRNAs play a complex role in tumorigenesis, drug resistance, and cancer therapy. Studies of miRNAs in GC still require more evidence at this time, as most studies have only looked at a small number of miRNAs in cell lines. In addition, support vector machine (SVM) is a robust machine learning method and is widely used in classification [6].

Although there are a large number of studies based on Cox and LASSO regression analysis to identify risk signatures, there are fewer studies on signature in GC patients with the SVM method. In order to obtain robust and stable results, we used SVM and Cox regression analysis to construct a miRNA signature. In conclusion, the construction of a novel miRNA signature is critical for the prognosis prediction of GC patients with the goal of exploring potential effects of miRNAs on immunotherapy, TME, biological processes, and MSI status.

## 2. Materials and Methods

### 2.1. Datasets

The Cancer Genome Atlas (TCGA) database was searched for transcriptome data and included 45 normal samples and 389 GC samples. The UCSC Xena Browser contains clinical data on TCGA-GC. Their survival information, clinicopathology, and genetics were retrieved and analyzed further. TCGA-GC cohort was randomly divided into 1 : 1 and represented as training set and testing set. The RNA-seq transcriptome data in CPM format and corresponding clinical data of GC patients were extracted from the database, and only genes with CPM greater than 1 were considered.

### 2.2. Calculation of Risk Score

Clinical data from GC cases in TCGA cohort were used to screen prognostic miRNAs linked with OS using univariate Cox regression analysis, and support vector machine (SVM) was used to eliminate redundant miRNAs. We selected miRNAs with *p* value less than 0.05 to undertake multivariate Cox regression analysis. The expression levels of the miRNAs and coefficients were then used to construct risk signature. The following formula was used to calculate the risk score for each patient:

(1) $$\begin{array}{c} \overset{n}{\underset{i=1}{\sum }}{\text{Coef}}_{i}*{\text{miRNA}}_{i}. \end{array}$$

We calculated the median score in TCGA-GC cohort to divide patients into two groups and identify the most significant differences in prognosis between the risk subgroups. To analyze the prediction performance of prognostic features on overall survival, Kaplan-Meier survival curves and ROC curves were used.

### 2.3. Biological Function Analysis

Differential expression analysis (mRNAs and miRNAs) was performed using the limma package. TargetScan, miRTarBase, and miRDB tools were used to screen out target mRNAs. We overlapped target mRNAs and differential expression mRNAs. Finally, the above genes were analyzed for gene enrichment.

### 2.4. Comprehensive Analysis

We used ssGSEA, XCELL, TIMER, QUANTISEQ, MCPCOUNT, EPIC, CIBERSORT, CIBERSORT-ABS, ESTIMETA, and TIDE algorithms for estimating the abundance of immune cells, immune-related pathway, immunotherapeutic response, and microsatellite instability (MSI) status. *p* values and Pearson correlation coefficients were obtained based on the study. Immune checkpoint-related gene and human leukocyte antigen (HLA) gene expression levels may be linked to immune checkpoint inhibitor therapy response. We explored the difference in gene expression levels between the two groups.

## 3. Results

### 3.1. Calculation of Risk Score in GC Patients

**Figure 1:**
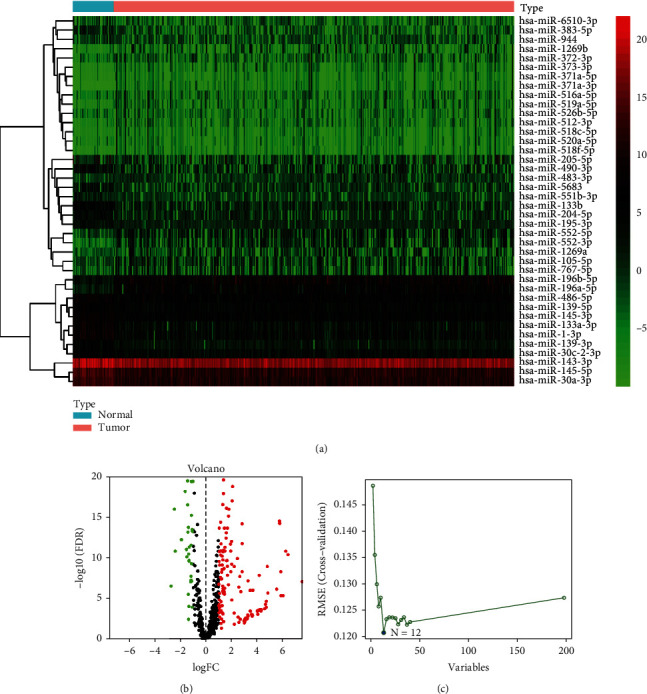
Screening of miRNAs: (a) the heat map of different expression miRNAs (DEmiRNAs); (b) volcano plot of DEmiRNAs; (c) the results of SVM.

**Figure 2:**
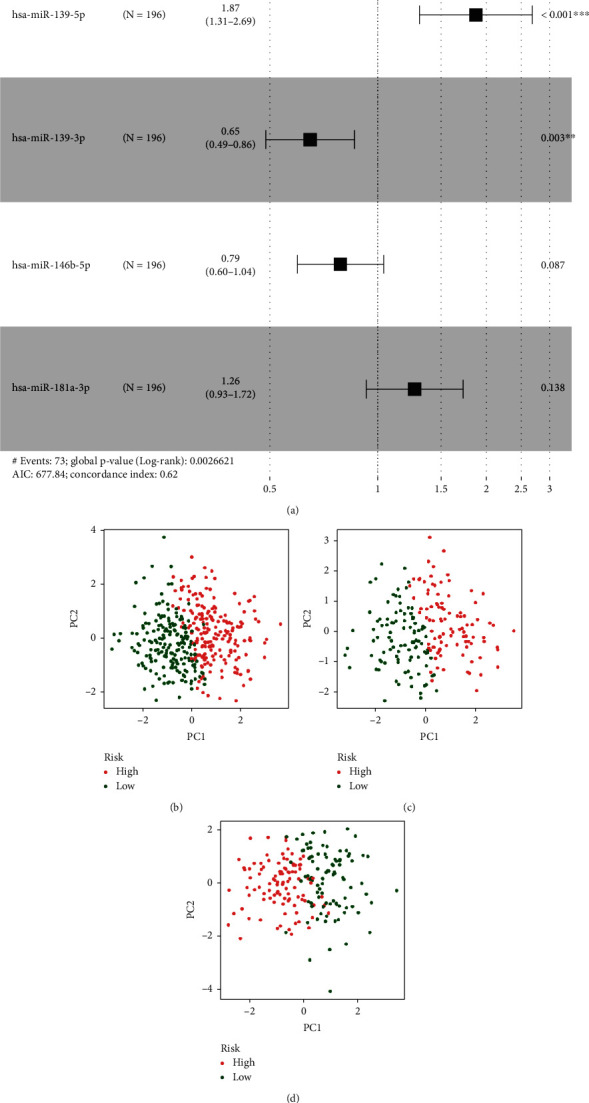
Construction of risk model. (a) A forest plot for results of multivariate Cox regression. PCA analysis of the entire cohort (b), training cohort (c), and testing cohort (d).

Using 45 normal samples as a control, we revealed that 138 were upregulated miRNAs and 60 were downregulated miRNAs. The volcano plot and heat map showed the 198 miRNA expression landscape (Figures 1(a) and 1(b)). In addition, SVM was used to screen robust 18 miRNAs in the above miRNAs (Figure 1(c)). Subsequently, multivariate Cox regression analysis was applied to 18 miRNA expression data in the training cohort for avoiding overfitting (Figure 2(a)). In detail, the risk score was determined according to the coefficients of each miRNA in the result of multivariate Cox regression analysis, and the formula is as follows: risk score = hsa − miR − 139 − 5p expression × 0.6271 + hsa − miR − 139 − 3p expression × −0.4345 + hsa − miR − 146b − 5p expression × −0.2398 + hsa − miR − 181a − 3p expression × 0.2347. According to the above formula, the risk score of each patient in TCGA-GC cohort was calculated. Subsequently, based on the median of risk score in the training cohort, we divided patients into two risk subgroups. PCA analysis showed that all samples from the different risks could be well distinguished in the entire cohort (Figure 2(b)), training cohort (Figure 2(c)), and testing cohort (Figure 2(d)).

### 3.2. Exploring Prognostic Value of Risk Score

**Figure 3:**
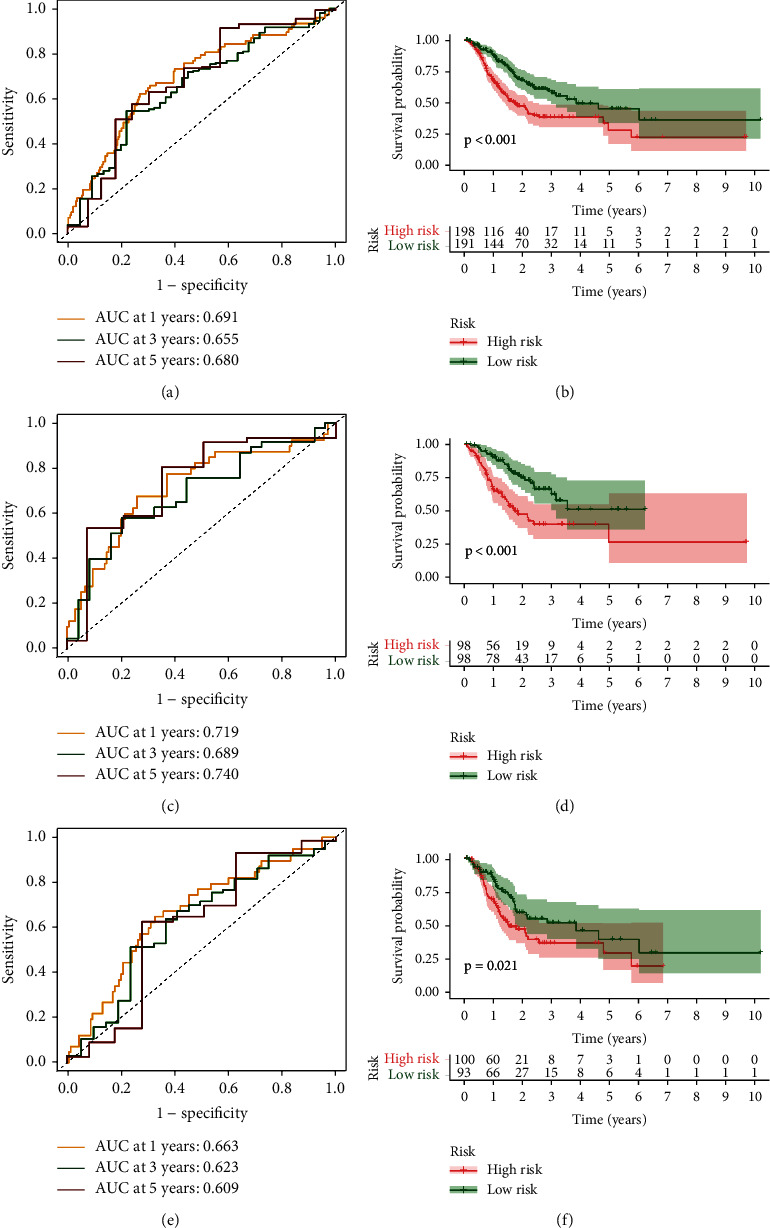
Prognostic value of risk score. ROC analysis of the entire cohort (a), training cohort (c), and testing cohort (e). Kaplan-Meier survival analysis of the entire cohort (b), training cohort (d), and testing cohort (f).

**Figure 4:**
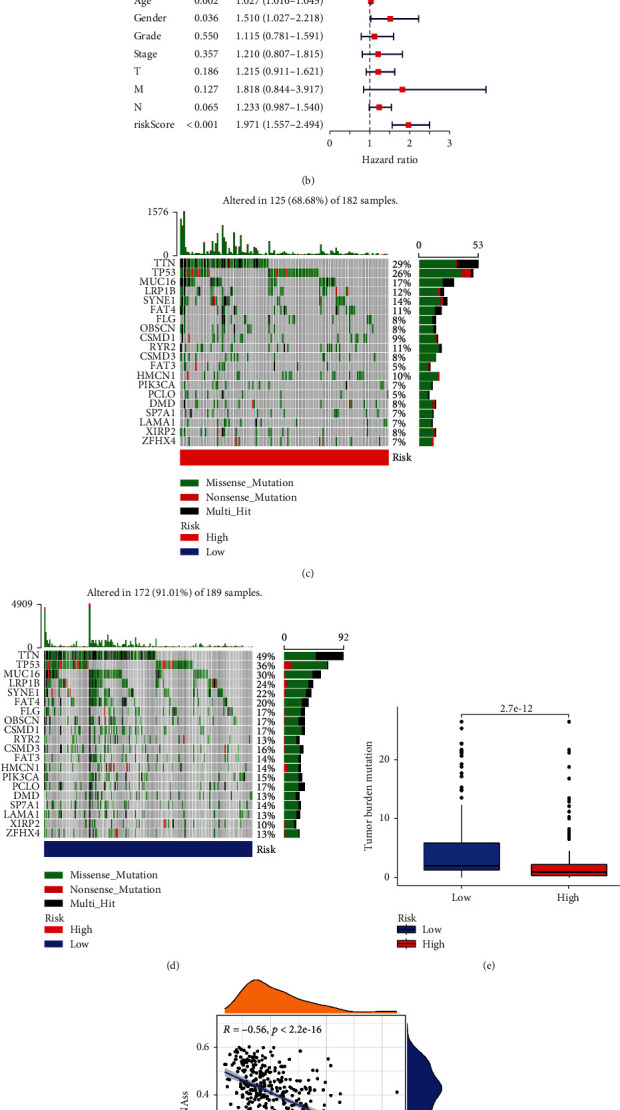
Somatic mutation analysis: (a) forest plot of univariate Cox regression analysis; (b) forest plot of multivariate Cox regression analysis; (c) somatic mutation analysis in the high-risk group; (d) somatic mutation analysis in the low-risk group; (e) analysis of differences in TMB in different risk groups; (f) correlation analysis between RNAs and risk score.

To better evaluate the prognostic value of risk score, we performed ROC analysis, and the AUC value showed a robust prediction accuracy (AUC > 0.7) in each cohort, as shown in Figures 3(a), 3(c), and 3(e). Meanwhile, the Kaplan-Meier analysis and log-rank test were used to estimate the predictive ability of the model for the clinical outcomes of GC patients (*p* < 0.05). The results showed that the OS of patients with low risk was better than those of high-risk patients in the entire cohort (Figure 3(b)), training cohort (Figure 3(d)), and testing cohort (Figure 3(f)). To determine whether risk score is an independent prognostic factor in GC patients, we included risk score and other clinical parameters in Cox regression analyses. Excitingly, univariate and multivariate Cox regression analyses suggested that risk score is a risk factor (Figure 4(a)) and an independent prognostic factor (Figure 4(b)). Specifically, in univariate and multivariate regression, HR value of risk score is 1.726 and 1.971, respectively (*p* < 0.001).

### 3.3. Somatic Mutation and Cell Stemness Analysis

We further analyzed the relationship between risk score and somatic mutation. The waterfall plot showed that patients in the low-risk group exhibited a wider range of mutations (Figures 4(a) and 4(b)). However, in different risk groups, TTN, TP53, MUC16, and LRP1B were the major mutation genes. In addition, the boxplot showed that low-risk patients have a higher TMB score (Figure 4(c)). Considering the effect of cell stemness on prognosis, we also analyzed cell stemness of patients with risk score and found that they were negatively related (Figure 4(d)).

### 3.4. Comprehensive Evaluation of Immune Function by Multiple Algorithms

**Figure 5:**
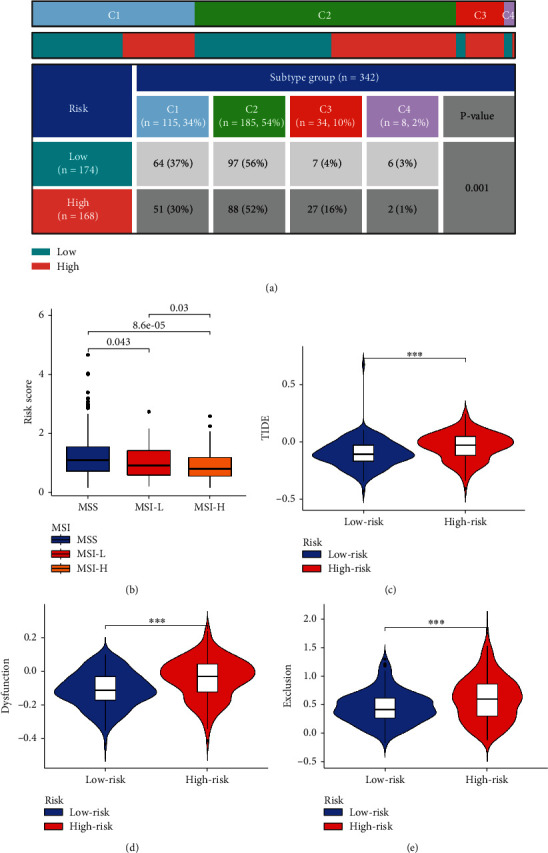
MSI status and immune subtype analysis: (a) distribution of immune subtypes and risk subgroups; (b) analysis of differences in TMB in different risk groups; (c) analysis of differences in MSI status, TIDE, dysfunction, and exclusion in different risk groups.

**Figure 6:**
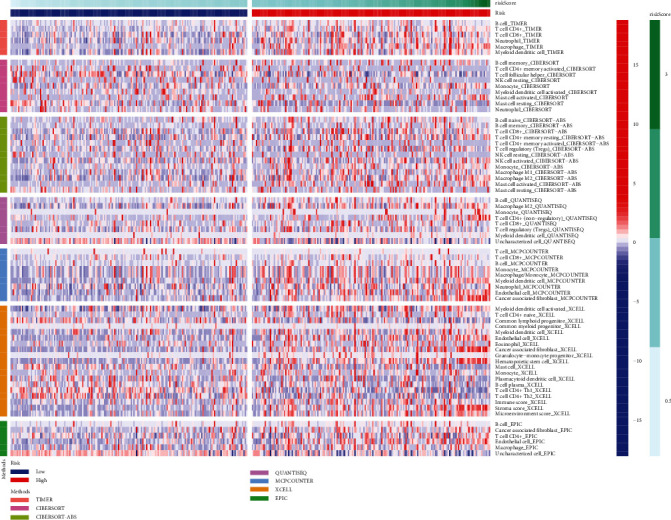
Difference analysis of immune cells and risk subgroups.

**Figure 7:**
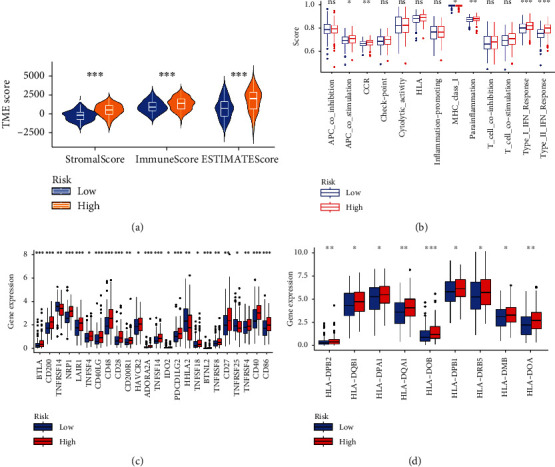
Immune function and immune checkpoint analysis in risk subgroups: (a) estimate analysis in different risk subgroups; (b) differential analysis of immune function; (c) differential expression analysis of immune checkpoint-related genes; (d) differential expression analysis of HLA-related genes.

We performed ANOVA for different risk patients and immune subtypes, and the differences were statistically significant (Figure 5(a)). We also analyzed microsatellite instability (MSI) of patients with different risks and found that the MSI-H grouping has a lower risk score (Figure 5(b)). The above results provide another potential explanation for the poor prognosis of patients with high risk score. Based on tumor pretreatment expression profiles, this TIDE module can estimate multiple published transcriptomic biomarkers to predict patient response [7]. In our risk subgroups, the high-risk group had higher TIDE score (Figure 5(c)), dysfunction score (Figure 5(d)), and exclusion score (Figure 5(e)) than the low-risk group. Moreover, XCELL, TIMER, QUANTISEQ, MCPCOUNT, EPIC, CIBERSORT, and, CIBERSORTABS algorithms were used to evaluate the content and correlation of immune infiltrating cells in different risk groups. In the person correlation analysis, we found that most of the immune cells calculated by 6 algorithms were negatively correlated with the risk score. In the difference analysis, B cell and T cell showed significant differences in most algorithm results, as shown in Figure 6. Subsequently, we explored the tumor microenvironment using the ESTIMATE algorithm, and we found that the high-risk group had higher estimate score, immune score, and stromal score compared with the low-risk group (Figure 7(a)). The ssGSEA algorithm also suggested that there are also significant differences in immune function between the different risk groups, including APC costimulation, CCR, MHC class I, parainflammation, and IFN response (Figure 7(b)). We selected 46 immune checkpoints commonly used in treatment, and the results showed that 24 immune checkpoints were significantly different between patients in the high- and low- risk groups (Figure 7(c)). Interestingly, immunofunctional analysis confirmed significant differences in HLA-related genes between the low-risk and high-risk groups in TCGA and GEO cohorts, as shown in Figure 7(d).

### 3.5. miRNAs Participating in Signature May Be Revolved in DNA Repair and Replication

**Figure 8:**
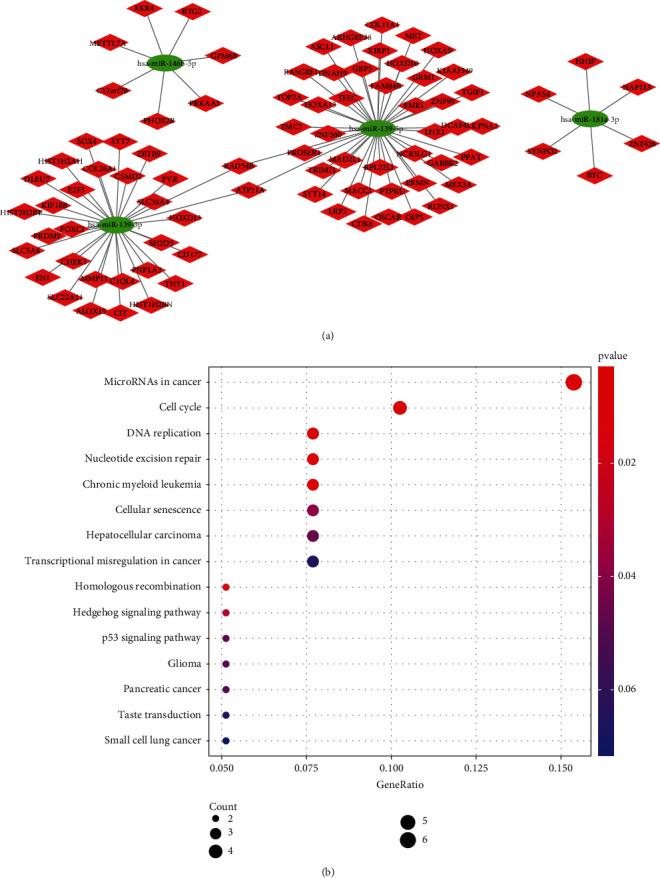
miRNA-mRNA network: (a) miRNA-mRNA network; (b) a bubble plot for the results of KEGG analysis.

TargetScan, miRTarBase, and miRDB tools were used to screen out target mRNAs in 4 miRNAs participating in signature. Subsequently, we overlapped target mRNAs and differential expression mRNAs. Finally, a potential functional regulation network is constructed (Figure 8(a)). To better understand the underlying molecular mechanisms and functions of the above mRNAs, interestingly, in KEGG analysis, we found that the above mRNAs were associated with DNA repair, DNA replication, and homologous recombination (Figure 8(b)).

## 4. Discussion

Gastric cancer (GC) is widely regarded as one of the most common malignant tumors of the digestive system, with high morbidity and mortality, and has attracted more and more attention. A series of discoveries of miRNAs have made significant progress in the field of cancer, especially in immune [8]. In recent years, a number of critical discoveries have highlighted the growing interest in understanding the mechanisms of miRNAs. And with the development of artificial intelligence, more new tools have been applied in the life sciences [9, 10]. However, the specific role and prognostic value of miRNAs in GC have not been clearly elucidated. In this study, using SVM-Cox model, we were able to construct a risk score formula based on 4 miRNAs. The training and testing cohorts were used to validate the performance of the risk score that was made. We also used the KEGG enrichment analyses to investigate the function of these miRNAs. XCELL, TIMER, QUANTISEQ, MCPCOUNT, EPIC, CIBERSORT, and, CIBERSORTABS algorithms were used to evaluate the content and correlation of immune infiltrating cells in different risk groups. The findings of the study imply that risk score has a significant impact on survival risk in GC patients and could be used as biomarkers for therapeutic targets.

The four miRNAs involved in the modeling have been studied in gastric cancer. For example, SNHG3 functions in an oncogenic manner to drive GC proliferation, migration, and invasion by regulating the miR-139-5p/MYB axis [11]. In addition, circ-PTPDC1 promotes the proliferation, migration, and invasion of GC cell lines via sponging miR-139-3p by regulating ELK1 [12]. In the current study, quantitative analyses revealed that the high-risk group had a higher percentage of immune-related cells and functions. Previous research has demonstrated that ferroptosis can emit damage-associated molecular or lipid mediators that attract antigen-presenting cells, triggering a cascade of innate and adaptive immune responses [13]. Consistent with previous studies, our study also shows that cells and functions associated with the antigen presentation process are significantly activated in the low-risk group, particularly in T cells and B cells [14]. It is also noteworthy that both T cells and B cells have a significant effect on the effect of antitumor activity of OSCC. Furthermore, ferroptosis combined with immune checkpoint inhibitors (ICIs) synergistically enhance antitumor activity, even in ICI-resistant types [12]. We identified several immune checkpoints that may guide our future targeted therapy options in OSCC patients, such as CD27, CD276, CD40, CD44, LAG3, LIGIT, TMIGD2, and TNFSF15. We found that many types of immune cells are different in different risk groups. In the current study, CD8^+^ T lymphocytes have also been demonstrated to cause lipid peroxidation in cancer cells and make cells more susceptible to ferrogenesis by releasing IFN [15]. As a result, we believe that further research into the involvement of these immune cells in ferroptosis and immune evasion is required in the future. Finally, based on the findings of this study, we can speculate that the poorer prognosis in the high-risk group may be due to dysregulation of antitumour immune function, which raises a more in-depth question: whether the development of GC can be caused by miRNA imbalance affecting antitumour immune function.

In conclusion, this study utilized comprehensive bioinformatics to analyze and establish 4-miRNA risk score formula, including hsa-miR-139-5p, hsa-miR-139-3p, hsa-miR-146b-5p, and hsa-miR-181a-3p, ultimately to identify potential biomarkers for predicting GC progression. And further analysis and study finally revealed the functions and mechanisms of these miRNAs. Due to the small sample size of the control group used for miRNA analysis in this study, only limited data can be presented in the paper. In summary, our data need to be further investigated and validated in a larger patient population and explored in future research together.

## Data Availability

Data is available at TCGA database (https://portal.gdc.cancer.gov/).
